# *Mycobacterium tuberculosis* Infection in School Contacts of Tuberculosis Cases: A Systematic Review and Meta-Analysis

**DOI:** 10.4269/ajtmh.23-0038

**Published:** 2024-04-23

**Authors:** Wenjin Wang, Aohan Liu, Xinjie Liu, Nannan You, Zhan Wang, Cheng Chen, Limei Zhu, Leonardo Martinez, Wei Lu, Qiao Liu

**Affiliations:** ^1^ Department of Chronic Communicable Disease, Center for Disease Control and Prevention of Jiangsu Province, Nanjing, People’s Republic of China;; ^2^ Center for Disease Control and Prevention of Yancheng City, Yancheng, People’s Republic of China;; ^3^ Department of Epidemiology, Mailman School of Public Health, Columbia University, New York, New York;; ^4^ Department of Epidemiology, School of Public Health, Shandong University, Jinan, People’s Republic of China;; ^5^ Department of Medical Records and Statistics, The Second Affiliated Hospital of Zhejiang Chinese Medical University, Hangzhou, People’s Republic of China;; ^6^ Department of Epidemiology, School of Public Health, Boston University, Boston, Massachusetts

## Abstract

Substantial tuberculosis transmission occurs outside of households, and tuberculosis surveillance in schools has recently been proposed. However, the yield of tuberculosis outcomes from school contacts is not well characterized. We assessed the prevalence of *Mycobacterium tuberculosis* infection among close school contacts by performing a systematic review. We searched PubMed, Elsevier, China National Knowledge Infrastructure, and Wanfang databases. Studies reporting the number of children who were tested overall and who tested positive were included. Subgroup analyses were performed by study location, index case bacteriological status, type of school, and other relevant factors. In total, 28 studies including 54,707 school contacts screened for *M. tuberculosis* infection were eligible and included in the analysis. Overall, the prevalence of *M. tuberculosis* infection determined by the QuantiFERON Gold in-tube test was 33.2% (95% CI, 0.0–73.0%). The prevalences of *M. tuberculosis* infection based on the tuberculin skin test (TST) using 5 mm, 10 mm, and 15 mm as cutoffs were 27.2% (95% CI, 15.1–39.3%), 24.3% (95% CI, 15.3–33.4%), and 12.7% (95% CI, 6.3–19.0%), respectively. The pooled prevalence of *M. tuberculosis* infection (using a TST ≥5-mm cutoff) was lower in studies from China (22.8%; 95% CI, 16.8–28.8%) than other regions (36.7%; 95% CI, 18.1–55.2%). The pooled prevalence of *M. tuberculosis* infection was higher when the index was bacteriologically positive (43.6% [95% CI, 16.5–70.8%] versus 23.8% [95% CI, 16.2–31.4%]). These results suggest that contact investigation and general surveillance in schools from high-burden settings merit consideration as means to improve early case detection in children.

## INTRODUCTION

Tuberculosis (TB) is a major cause of morbidity and mortality worldwide. Approximately 10% of the 9.9 million cases of TB were in children aged under 15 years globally in 2019.^1^^,^^2^ Children with recent *Mycobacterium tuberculosis* infection have an 8–20% risk to progress to TB disease 2 years after exposure.^3^^,^^4^ Despite this, many children at high risk of developing TB are not identified, screened, and given preventive therapy.^5^ Historically, children have been largely neglected in TB control efforts. To reduce morbidity and mortality of TB among children, a rigorous evaluation of distinct strategies for case detection and prevention is needed.

The most effective and appropriate TB control program directed at children and adolescents is debated. Whether to target specific high-risk children, settings of high risk, or a more broad-based implementation strategy is unclear. Prior studies have found household contact tracing to have a high yield and to be cost-effective in countries with high TB incidence settings.^6^^,^^7^ However, emerging evidence suggests that *M. tuberculosis* infections among children are often acquired outside the household, suggesting that complementary interventions and strategies in addition to household contact tracing are needed.^8^^,^^9^ Whether screening approaches in other settings, such as schools, have a substantial yield has not been systematically evaluated.

There has been no published review exploring the prevalence of *M. tuberculosis* infection among close school contacts of TB cases. We aimed to review the evidence on the yield of school contact investigations in distinct settings. We aimed to collate data from similar settings to provide information that can be used to estimate the benefit of such interventions. We conducted a systematic review and meta-analysis of the prevalence of *M. tuberculosis* infection in young school contacts of TB cases and by specific subgroups of these children.

## MATERIALS AND METHODS

### Study design and search strategy.

We conducted a systematic review of studies that included children as school contacts of a person with TB. We first searched the literature for systematic reviews investigating *M. tuberculosis* infection in school contacts of TB among children. None were found. We then aimed to compile all studies investigating children in close contact with a person with TB in the same school.

A literature search was performed using the PubMed, Elsevier, China National Knowledge Infrastructure, and Wanfang databases. The Medical Subject Headings keywords and terms “pulmonary tuberculosis,” “tuberculosis,” “TB,” “tuberculosis infection,” “latent tuberculosis infection,” “latent TB infection,” “tuberculin skin test,” “interferon-gamma release assays,” “IGRAs,” “QuantiFERON-TB Gold In-Tube,” “QFT,” “T-SPOT,” “students,” “child,” “children,” “adolescent,” “school,” “teenage,” “baby,” “juvenile,” “young,” “pupil,” “infant,” and “kid” were used. Boolean operators (AND and OR) were also used individually or in combinations to conduct a comprehensive search. All search terms were searched in titles, abstracts, and field keywords. Additionally, the search was restricted to papers published from database inception through January 1, 2022, without language restrictions. The literature search was performed by three authors (W. Wang, X. Liu, and A. Liu). Two authors (W. Wang and X. Liu) independently reviewed titles and then abstracts, in parallel and independently, for relevance and included publications identified by either author for full-text review. Two authors (W. Wang and A. Liu) extracted data on methods from included surveys using an electronic form and gathered datasets from supplemental materials.

### Inclusion and exclusion criteria.

The review included retrospective surveys, cross-sectional studies, and outbreak investigations. Systematic reviews, case reports, case series, editorials, and letters to the editors were excluded. We included studies that showed the prevalence of *M. tuberculosis* infection among school contacts under 18 years old and used the enzyme-linked immunosorbent assay and/or the enzyme-linked immunosorbent spot-based interferon gamma release assay (IGRA) and/or the tuberculin skin test (TST) with a 5-, 10-, or 15-mm induration diameter as the cutoff for positive results. Furthermore, the unavailability of full text and duplicate publications were also considered exclusion criteria. When we identified more than one study for a single survey, we included the earliest source or most complete dataset and excluded other records. All the processes were independently completed by two reviewers to decrease the risk of errors.

### Data extraction.

The data from each study were recorded in a data extraction form designed by the reviewers. The retrieved data included the first author’s name, year of publication, study location, study design, whether the study was an outbreak, sample size, age, sputum smear, school type, whether the study was a boarding school, bacillus Calmette-Guérin (BCG) vaccination, TST induration diameter cutoff, results of the etiological examination, and the number of *M. tuberculosis* infections among included children. The prevalence of *M. tuberculosis* infection was the percentage of *M. tuberculosis* infections among screened contacts.

## STATISTICAL ANALYSES

Data analysis was conducted using R statistical software (v. 4.1.2; The R Foundation for Statistical Computing, Vienna, Austria). Wilson’s method was used to calculate 95% CIs; the *I*^2^ statistic was used to determine heterogeneity.^10^^–^^12^ *I*^2^ values greater than 50% were considered to represent substantial heterogeneity; random-effects models were used when substantial heterogeneity was present. Effect sizes were reported as proportions. Subgroup analysis was performed based on country, study design, presence or absence of bacteriological positivity, level of schooling (primary, secondary, or high school), boarding school, and induration diameter cutoff (≥5, ≥10, and 15 mm).

## RESULTS

### Study selection.

**Figure 1.**
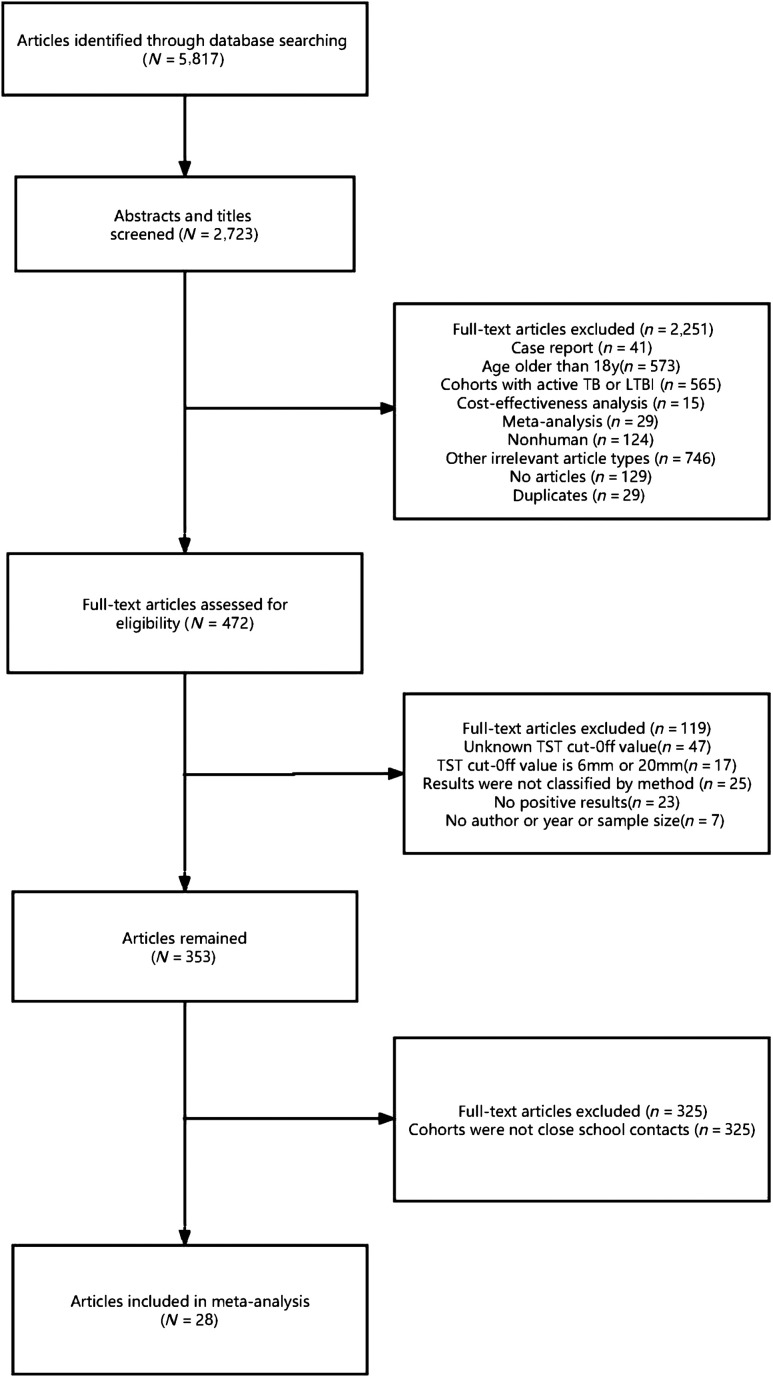
Flow chart of the study inclusion. LTBI = latent tuberculosis infection; TB = tuberculosis; TST = tuberculin skin test.

In total, 5,817 articles were reviewed for titles and abstracts, and 3,094 articles were excluded. Of the remaining 2,723 articles, 472 articles were eligible after full-text review. In the end, we identified 28 studies to be included in this meta-analysis that reported data of *M. tuberculosis* infection in school contacts under 18 years old. The flow diagram of the study selection process is shown in Figure 1.

### Study characteristics and prevalence of *Mycobacterium tuberculosis* infection.

The primary characteristics of the studies included in the review are summarized in Table 1. In total, 28 studies with 54,707 school contacts were included in the pooled prevalence of *M. tuberculosis* infection, of which 19 studies were from China,^13^^–^^31^ three were from Italy,^32^^–^^34^ two were from Korea,^35^^,^^36^ and four studies each were from Iran,^37^ Japan,^38^ Sweden,^39^ and the United States,^40^ respectively. In regard to study design, 15 were cross-sectional studies and 11 were retrospective studies. The proportions of students in high school, middle school, and primary school were 47.2% (25,805/54,707), 10.0% (5,471/54,707), and 3.2% (1,774/54,707), respectively. There were 39.3% (21,473/54,707) of unknown school type, and just 0.3% (184/54,707) were children in kindergarten. A total of 36.0% (19,712/54,707) of students were vaccinated with BCG, one study in which students were not vaccinated with BCG, and for 64.0% (34,994/54,707), the vaccination status was unknown.

**Table 1:** Characteristics of 28 selected studies measuring *Mycobacterium tuberculosis* infection in close school contacts

Reference, Year	Country	Study Design	No. of Initial Cases	Sputum Smear*	Age (years)	Level of School	Boarding School	BCG Vaccination (%)	Diagnostic Test Used and Cutoff	Total, *N*	Total with Outcome	Prevalence (%)
Fang et al.,^13^ 2021	China	Outbreak investigation	1	Positive	16–17	High school	No	100	TST ≥15 mm	405	30	7.4
You et al.,^14^ 2019	China	Outbreak investigation	2	ATB	17–20	High school^†^	Yes	100	TST ≥5 mm	845	44	5.2
									TST ≥10 mm	845	65	7.7
Kim et al.,^35^ 2018	Korea	Cross-sectional study	43	ATB	17–18	High school	No	65	TST ≥10 mm	947	282	29.8
									QFT, 0.35 IU/mL	947	258	27.2
Kim et al.,^36^ 2015	Korea	Retrospective study	NS	ATB	12–19	High school	NS	70	TST ≥10 mm	7,475	1,861	24.9
Wei et al.,^15^ 2020	China	Retrospective study	6	ATB	NS	NS	NS	NS	TST ≥5 mm	7,702	742	9.6
Jing et al.,^16^ 2020	China	Retrospective study	26	ATB	≥15	Primary school, middle school, high school	NS	NS	TST ≥5 mm	10,062	101	1.0
					<15					1,555	28	1.8
Ou et al.,^17^ 2020	China	Retrospective study	1	Positive	NS	Middle school	No	NS	TST ≥15 mm	1,753	70	4.0
						High school				1,046	32	3.1
Zhang,^18^ 2015	China	Retrospective study	5	ATB	NS	Primary school, middle school	NS	NS	TST ≥15 mm	524	41	7.8
Ding and Zhang,^19^ 2015	China	Cross-sectional study	2	ATB	NS	High school	No	NS	TST ≥5 mm	4,258	141	3.3
Weng and Wang,^20^ 2014	China	Retrospective study	34	ATB	NS	NS	No	NS	TST ≥5 mm	710	475	66.9
Wang et al.,^21^ 2019	China	Retrospective study	NS	ATB	NS	High school	NS	NS	TST ≥15 mm	193	13	6.7
Yuan et al.,^22^ 2014	China	Cross-sectional study	NS	ATB	NS	NS	NS	NS	TST ≥5 mm	802	572	71.3
Ma,^23^ 2019	China	Retrospective study	9	ATB	NS	Secondary school	No	NS	TST ≥5 mm	443	50	11.3
Chen et al.,^24^ 2018	China	Cross-sectional study	1	ATB	NS	Secondary school	NS	NS	TST ≥5 mm	841	132	15.7
Xu et al.,^25^ 2021	China	Cross-sectional study	4	ATB	NS	High school^†^	Yes	NS	TST ≥5 mm	462	134	29.0
Pu et al.,^26^ 2018	China	Cross-sectional study	1	Positive	NS	High school^†^	Yes	NS	TST ≥15 mm	506	48	9.5
Ma et al.,^27^ 2021	China	Retrospective study	11	ATB	NS	High school	NS	100	TST ≥5 mm	342	72	21.1
Hou et al.,^28^ 2020	China	Cross-sectional study	1	ATB	16–18	High school^†^	Yes	100	TST ≥15 mm	395	130	32.9
Filia et al.,^32^ 2011	Italy	Cross-sectional study	1	ATB	2–6	Kindergarten	NS	NS	TST ≥5 mm	184	19	10.3
					5–11	Primary school				199	24	12.1
Faccini et al.,^33^ 2013	Italy	Cross-sectional study	1	Positive	NS	Primary school	NS	NS	TST ≥5 mm	977	188	19.2
Baghaie et al.,^37^ 2012	Iran	Cross-sectional study	1	Negative	15	High school	NS	100	TST ≥10 mm	52	17	32.7
Fang et al.,^29^ 2013	China	Retrospective study	1	Positive	NS	High school	NS	100	TST ≥15 mm	476	122	25.6
Higuchi et al.,^38^ 2009	Japan	Cross-sectional study	1	Positive	8–12	Primary school	NS	100	TST ≥5 mm	306	200	65.4
									TST ≥10 mm	306	90	29.4
									QFT	308	6	2.0
Pan et al.,^30^ 2019	China	Retrospective study	117	ATB	NS	High school	NS	100	TST ≥5 mm	4,078	625	15.3
						Middle school			TST ≥5 mm	2,434	559	23.0
Müller et al.,^39^ 2008	Sweden	Cross-sectional study	1	ATB	6–15	Primary school	NS	100	TST ≥15 mm	261	35	13.4
Molicotti et al.,^34^ 2008	Italy	Cross-sectional study	1	Positive	10	Primary school	NS	NS	TST ≥5 mm	29	19	65.5
									QFT	29	21	72.4
Huang et al.,^31^ 2018	China	Cross-sectional study	NS	ATB	16–18	High school	NS	100	TST ≥15 mm	4,325	352	8.1
Adler-Shohet et al.,^40^ 2014	USA	Cross-sectional study	1	Positive	NS	NS	NS	NS	TST ≥5 mm	118	31	26.3

NS = not shown; QFT = QuantiFERON-TB Gold In-Tube; TST = tuberculin skin test.

* For sputum smears, we divided studies into two groups according to the bacteriological results of index tuberculosis patients: ATB = active tuberculosis included studies with no detailed information of the bacteriological results of cases; Positive = bacteriologically positive.

^†^ Boarding school.

**Figure 2.**
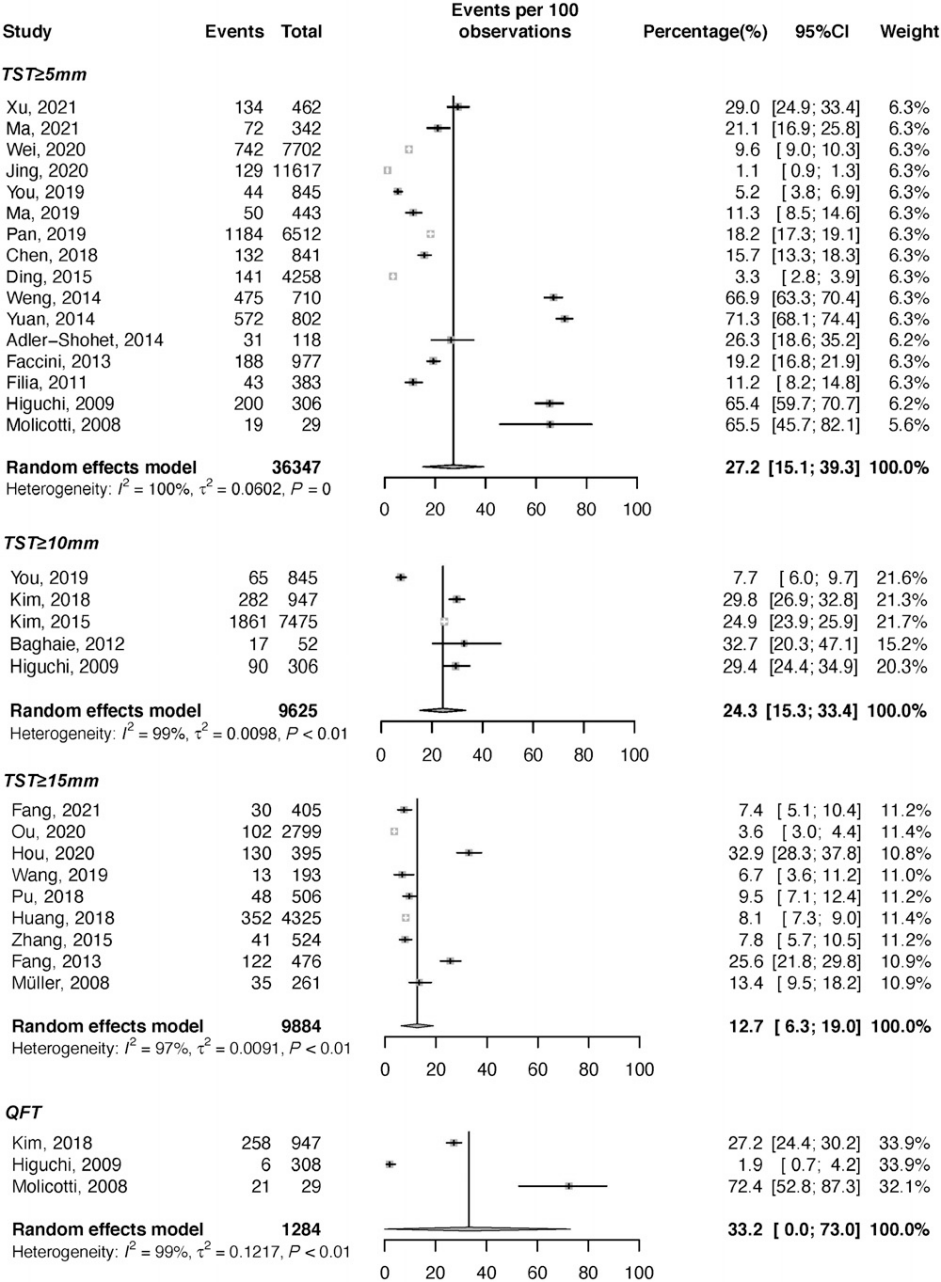
Forest plot of the prevalence of *Mycobacterium tuberculosis* infection based on distinct diagnostic tests and cutoffs. QFT = QuantiFERON-TB Gold In-Tube; TST = tuberculin skin test.

The vast majority of studies used TST screening, whereas only three studies used both the TST and the QuantiFERON-TB Gold In-Tube (QFT). There was high heterogeneity between studies; *M. tuberculosis* infection prevalence ranged from 1.1% to 72.4%. When a random-effects model was applied, we found that the pooled *M. tuberculosis* infection prevalences determined by the TST with induration cutoffs of ≥5 mm, ≥10 mm, and ≥15 mm and the QFT were 27.2% (95% CI: 15.1–39.3%, *I*^2^ = 100%), 24.3% (95% CI: 15.3–33.4%, *I*^2^ = 99%), 12.7% (95% CI: 6.3–19.0%, *I*^2^ = 97%), and 33.2% (95% CI: 0.0–73.0%, *I*^2^ = 99%), respectively (Figure 2). Twenty studies described the TB prevalence at baseline among contacts screened, and 11 studies described TB incidence during the follow-up time. The TB prevalence ranged from 0.0% to 15.0%, and the pooled TB prevalence among contacts screened was 0.7% (95% CI, 0.5–0.9%). The pooled TB incidence rate among contacts screened was 1.2% (95% CI, 0.8–1.6%), and ranged from 0.0% to 12.9% (Supplemental Table S1; Supplemental Figures 1 and 2).

### Percentage prevalences of *Mycobacterium tuberculosis* infection by location.

The pooled *M. tuberculosis* infection percentage, when a TST ≥5-mm induration cutoff was used, in studies from China was lower than that in studies from other regions (22.8%, 95% CI: 16.8–28.8%, versus 36.7%, 95% CI: 18.1–55.2%). The pooled *M. tuberculosis* infection percentage, based on a TST ≥15-mm cutoff, in China was 12.3% (95% CI: 8.4–16.1%, *I*^2^ = 97.7%). The pooled *M. tuberculosis* infection percentages detected by a TST ≥10-mm cutoff and QFT were 28.0% (95% CI: 24.3–31.8%, *I*^2^ = 77.3%) and 31.9% (95% CI: 9.0–54.7%, *I*^2^ = 99.3%), respectively, in other regions (Table 2; Supplemental Figure 3).

**Table 2:** Subgroup analysis of studies measuring *Mycobacterium tuberculosis* infection in close school contacts from a meta-analysis of 28 studies published

Stratum*	No. of Studies	Pooled Prevalence (%)	95% CI (%)	*I*^2^ (%)	References
All studies using different tests					
TST ≥5 mm	16	27.2	15.1–39.3	100	14–16,19,20,22–25,27,30,32–34,38,40
TST ≥10 mm	5	24.3	15.3–33.4	99	14,35–38
TST ≥15 mm	9	12.7	6.3–19.0	97	13,17,18,21,26,28,29,31,39
QFT	3	33.2	0.0–73.0	99	34,35,38
Locations					
China					
TST ≥5 mm	11	22.8	16.8–28.8	100	14–16,19,20,22–25,27,30
TST ≥15 mm	8	12.3	8.4–16.1	98	13,17,18,21,26,28,29,31
Other countries					
TST ≥5 mm	5	36.7	18.1–55.2	99	32–34,38,40
TST ≥10 mm	4	28.0	24.3–31.8	77	35–38
QFT	3	31.9	9.0–54.7	99	34,35,38
Case types					
Bacteriologically positive TB					
TST ≥5 mm	4	43.6	16.5–70.8	99	33,34,38,40
TST ≥15 mm	4	11.3	4.2–18.5	98	13,17,26,29
Active TB					
TST ≥5 mm	11	23.8	16.2–31.4	100	14,15,19,20,22–25,27,30,32
TST ≥10 mm	3	20.8	8.3–33.2	99	14,35,36
TST ≥15 mm	5	13.5	7.5–19.5	96	18,21,28,31,39
Levels of school					
Primary school					
TST ≥5 mm	4	39.8	15.7–63.9	99	32–34,38
Secondary school					
TST ≥5 mm	2	19.4	12.3–26.5	96	24,30
High school					
TST ≥5 mm	5	14.5	7.4–21.6	99	14,19,25,27,30
TST ≥10 mm	3	27.7	23.2–32.2	81	35–37
TST ≥15 mm	7	12.9	8.3–17.6	81	13,17,21,26,28,29,31

QFT = QuantiFERON-TB Gold In-Tube; TB = tuberculosis; TST = tuberculin skin test.

* The induration cutoff is shown with TST.

### Percentage prevalences of *Mycobacterium tuberculosis* infection by index TB patient characteristics.

We divided studies into three groups according to the bacteriological results of index TB patients. The group of “active TB” included studies that had no detailed information of the bacteriological results of cases. *M. tuberculosis* infection percentages in contacts with bacteriologically positive TB (43.6%, 95% CI: 16.5–70.8%, *I*^2^ = 98.8%) was higher than that in contacts with active TB (23.8%, 95% CI: 16.2–31.4%, *I*^2^ = 99.7%) when a TST induration cutoff of ≥5 mm was used. The percentage prevalence of *M. tuberculosis* infection in contacts with bacteriologically positive TB (11.3%, 95% CI: 4.2–15.8%, *I*^2^ = 97.8%) was similar to that in contacts with active TB (13.5%, 95% CI: 7.5–19.5%, *I*^2^ = 96.5%) when a TST cutoff of ≥15 mm was used (Table 2; Supplemental Figure 4).

### Percentage prevalences of *Mycobacterium tuberculosis* infection by school grade.

The pooled *M. tuberculosis* infection percentages detected using a TST cutoff of ≥5 mm in high school, secondary school, and primary school were 14.5% (95% CI: 7.4–21.6%, *I*^2^ = 99.3%), 19.4% (95% CI: 12.3–26.5%, *I*^2^ = 95.7%), and 39.8% (95% CI: 15.7–63.9%, *I*^2^ = 99.0%), respectively. The analysis revealed that the overall percentage prevalences of *M. tuberculosis* infection were 27.7% (95% CI: 23.2–32.2%, *I*^2^ = 81.7%) in high school students when a TST cutoff of ≥10 mm was used and 12.9% (95% CI: 8.3–17.6%, *I*^2^ = 97.8%) in high school students when a TST cutoff of ≥15 mm was used. Only one study of high school students and no study of secondary school students were performed to calculate *M. tuberculosis* infection percentage prevalences detected using QFT (Table 2; Supplemental Figure 5).

### Percentage prevalences of *Mycobacterium tuberculosis* infection by boarding school.

We divided studies into two groups, one which focused on studies of boarding schools and one which focused on studies that did not. The pooled percentage prevalence of *M. tuberculosis* infection, based on the results of a TST induration cutoff of ≥5 mm, was 17.2% (95% CI: 0.0–39.7%, *I*^2^ = 98.6%) in boarding schools. A TST cutoff of ≥5 mm was not used in any of the included studies to calculate the percentage prevalence of *M. tuberculosis* infection among nonboarding school students. QFT was not used in any of the included studies to calculate the prevalence of *M. tuberculosis* infection among boarding school students or nonboarding school students.

## DISCUSSION

To our knowledge, this is the first study to systematically collect and analyze the prevalence of *M. tuberculosis* infection in school contacts of TB cases. *M. tuberculosis* infection among school contacts was high overall but largely varied from 27.2% to 24.3% to 12.7% when TST cutoffs of 5 mm, 10 mm, and 15 mm were used. The prevalence of *M. tuberculosis* infection was highest (33.2%) among children from studies that used QFT tests. These study results show that the prevalence is substantially higher than the background prevalence of *M. tuberculosis* infection among young children,^41^^–^^44^ suggesting that considerable transmission is occurring among school contacts of TB cases, representing an important population for targeted intervention.

Surprisingly, we found a lower prevalence of *M. tuberculosis* infection in studies from China than in those from other countries. This remained the case regardless of the TST induration cutoff used to define *M. tuberculosis* infection. The reason for this result is not immediately clear but may be related to BCG vaccination. In China, all newborns were BCG vaccinated due to a strong immunization program.^45^ BCG vaccination has been found to protect against both *M. tuberculosis* infection and TB disease in young children.^46^^,^^47^ The strong BCG vaccination coverage in China may partially explain this result by protecting young school contacts in these studies. Alternatively, many of the studies outside of China included bacteriologically positive index cases. Bacteriological status was often not mentioned in studies from China. This may also explain the lower *M. tuberculosis* infection prevalence in studies from China.

Our results suggest a high prevalence of *M. tuberculosis* infection among school contacts irrespective of school grade. But it is worth noting that primary school contacts may have a higher likelihood of developing *M. tuberculosis* infection, and the prevalence reached 40% when a TST induration cutoff of ≥5 mm was used. A meta-analysis involving children with *M. tuberculosis* infection in Europe, the United States, Asia, and Africa showed that the infection rate of *M. tuberculosis* infection children with close family contact with TB was nearly four times that of children without close family contact.^48^^,^^49^ These studies indicate that children are the high-risk group for *M. tuberculosis* infection and that preventive measures for young children are urgently needed.

Additionally, our study revealed that there was little difference in prevalence of *M. tuberculosis* infection between boarding and nonboarding schools, implying that boarding school attendance may not be an important factor in *M. tuberculosis* infection. One study indicated a higher risk of *M. tuberculosis* infection in boarding schools (19%) than day schools (5%)^9^; ventilation and time spent in the classroom may play an important role in TB transmission and acquisition.^9^^,^^50^ However, there were too few boarding school studies in our study, potentially impacting the final result.

This review has several limitations. First, less information was available among index cases from included studies, limiting our ability to understand how disease severity impacted subsequent transmission. We recommend that future social contact surveys collect and report school-based data, ideally using standardized tools to indicate the bacteriological status of TB cases. Second, substantial between-study variability was present. Enduring heterogeneity suggests that secondary factors that were not collected (or not collected systematically by all studies) may have impacted this analysis. These may include undocumented secondary exposures among school contacts, reliability of the diagnostic tests performed,^51^ and BCG vaccination status, among others. Third, most studies from China used a TST induration cutoff of ≥15 mm to define *M. tuberculosis* infection; differential classification of *M. tuberculosis* infection status by study and region may have impacted our pooled results. Fourth, we included only studies that evaluated *M. tuberculosis* infection immunologically, and results would differ based on different screening methods. Thus, our conclusion may not generalize to some regions that lack the ability to screen TB infection by IGRA or TST. Generally, these regions have a high burden of TB, so our results may underestimate TB infection among school contacts.

## CONCLUSION

In summary, we found a high prevalence of *M. tuberculosis* infection among school contacts. The excess burden of TB among school contacts has serious implications for *M. tuberculosis* transmission. High between-study heterogeneity suggests that local transmission dynamics are critical for understanding high-transmission locations. However, if confirmed, an integrated and consistent facility-based and community-based effort addressing prevention, early detection, and management of *M. tuberculosis* infection should be further investigated and strengthened for control of TB among young children.

## Supplemental Materials

10.4269/ajtmh.23-0038 Supplemental Materials

## Data Availability

Please contact W. Wang for data requests.
