# Supplementary material for: Mycobacterium tuberculosis Infection in School Contacts of Tuberculosis Cases: A Systematic Review and Meta-Analysis
Source: Am J Trop Med Hyg. 2024 Apr 23;110(6):1253–60. doi: 10.4269/ajtmh.23-0038 (PMC11154035; doi:10.4269/ajtmh.23-0038)
Supplement: Supplemental Materials [file tpmd230038.SD1.pdf]

Supplementary Figure 1 (Flow chart)

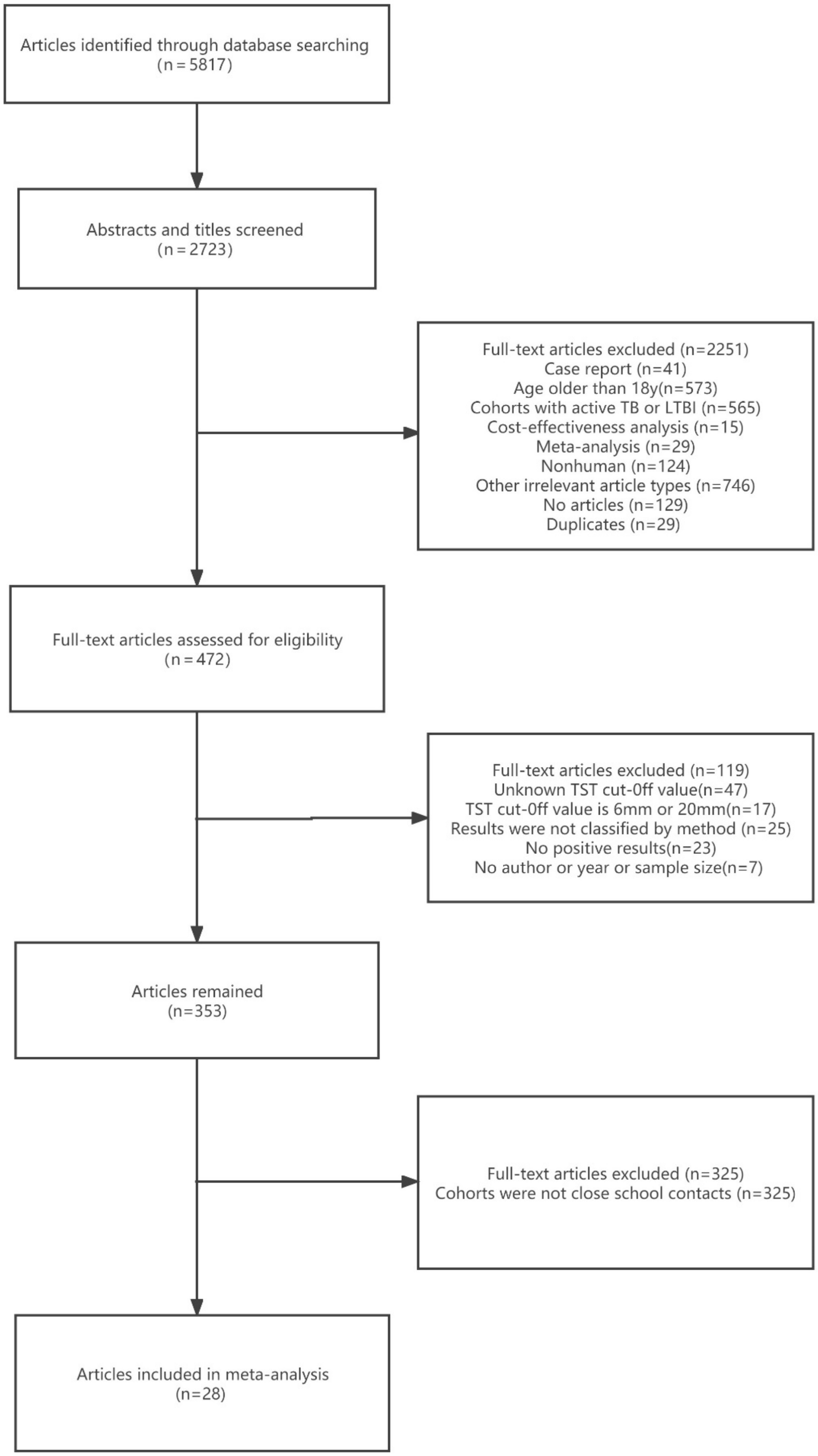

Supplementary Figure 1

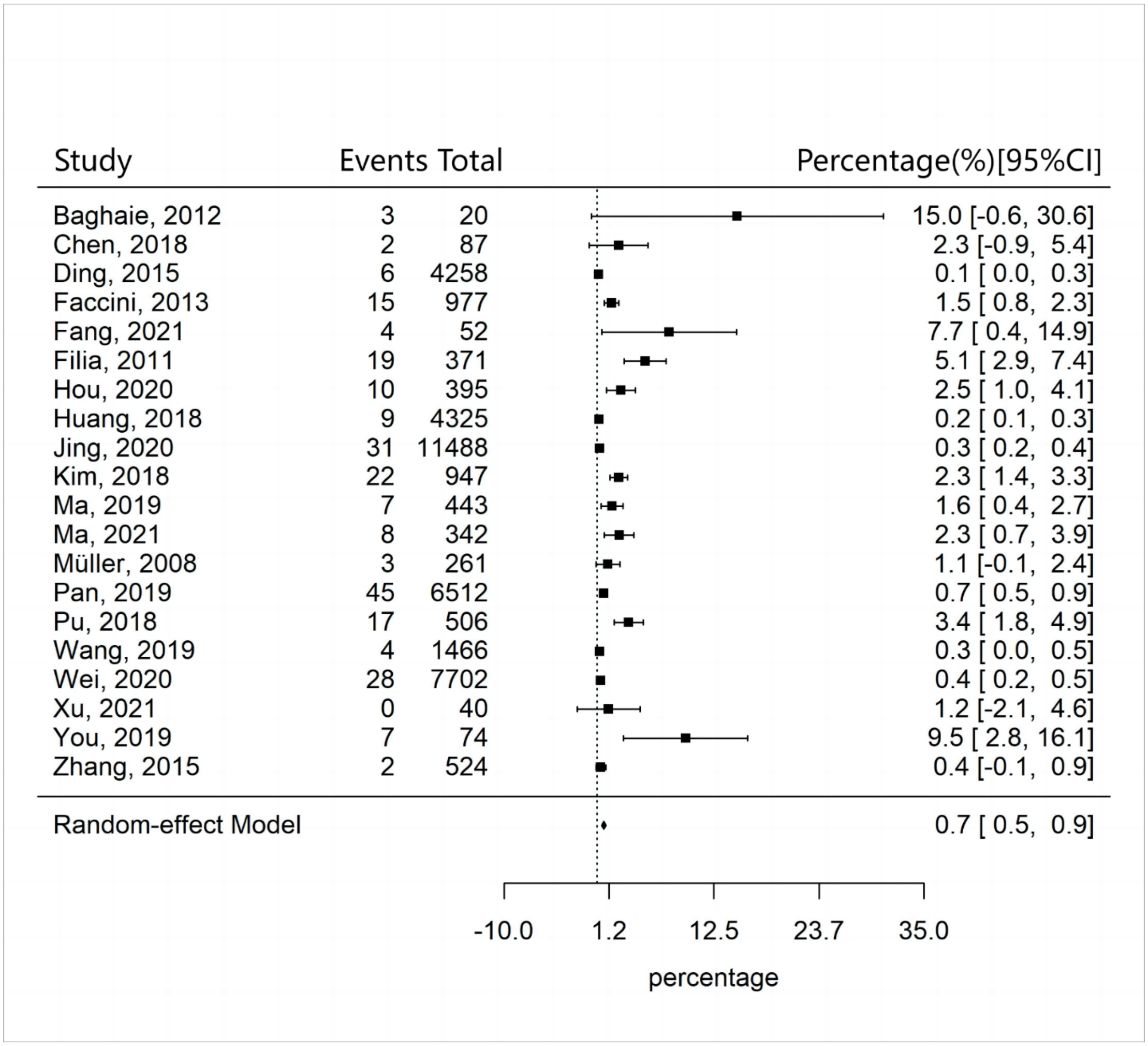

Supplementary Figure 2 (Forest plot)

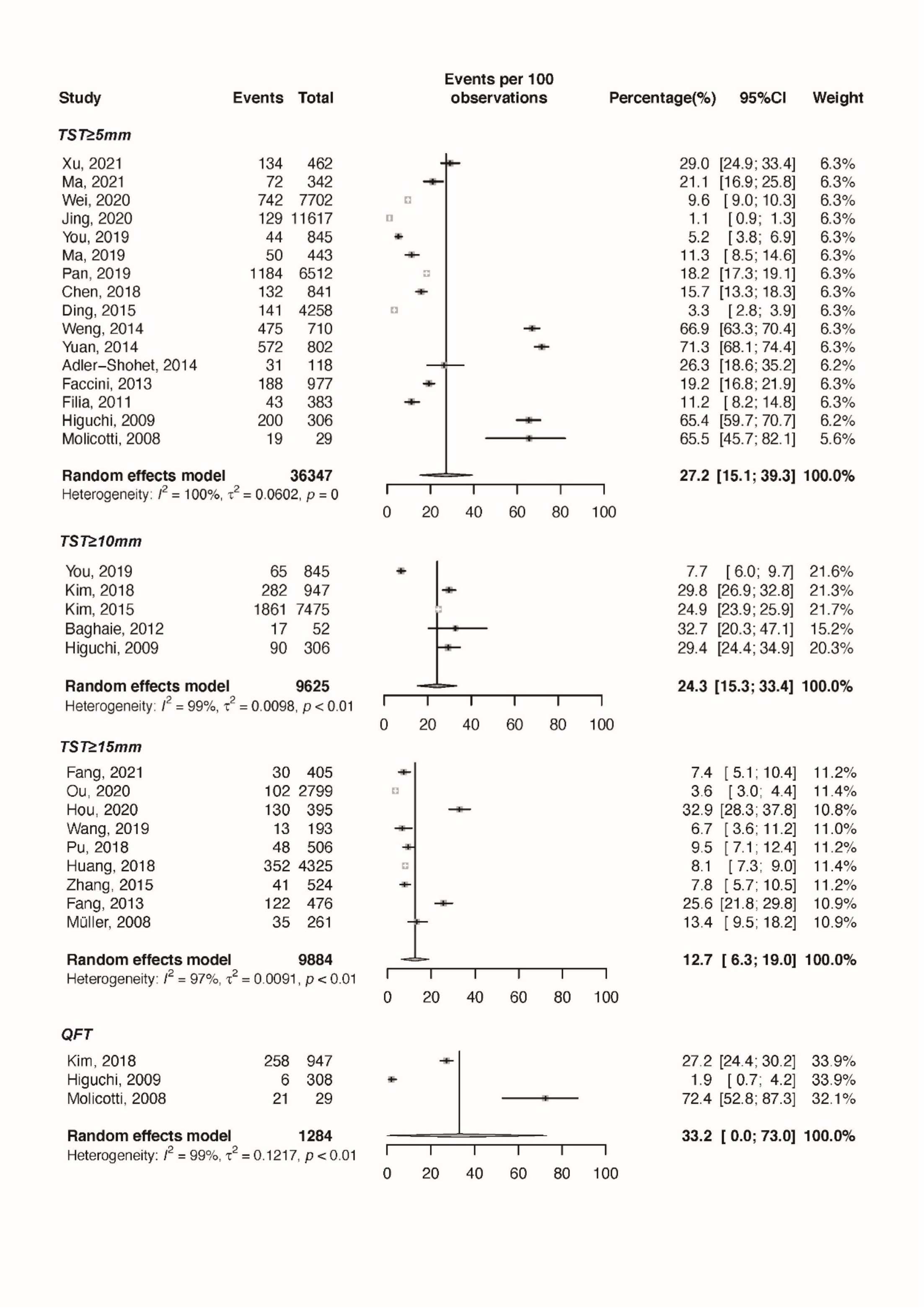

Supplementary Figure 2

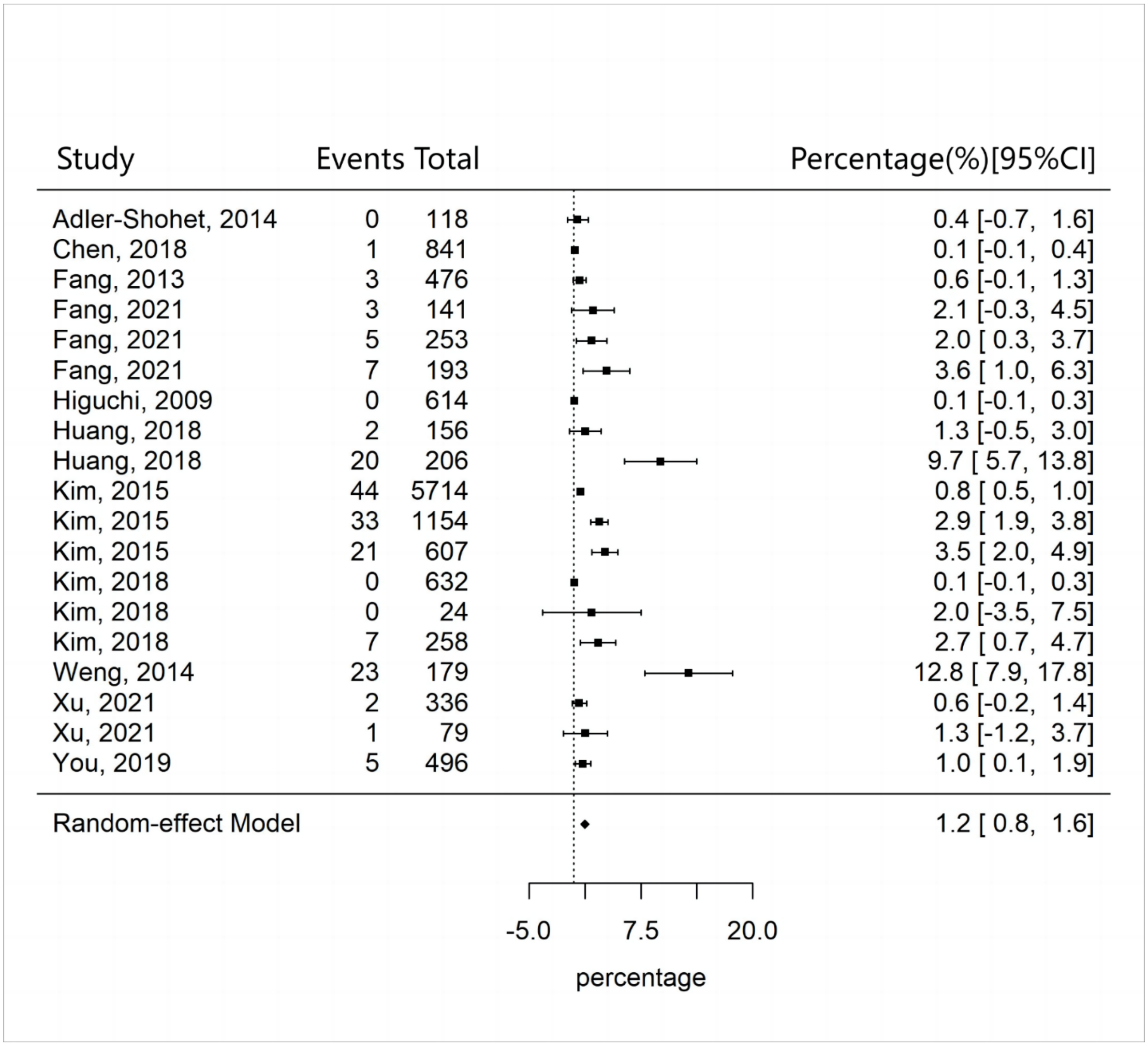

Supplementary Figure 3

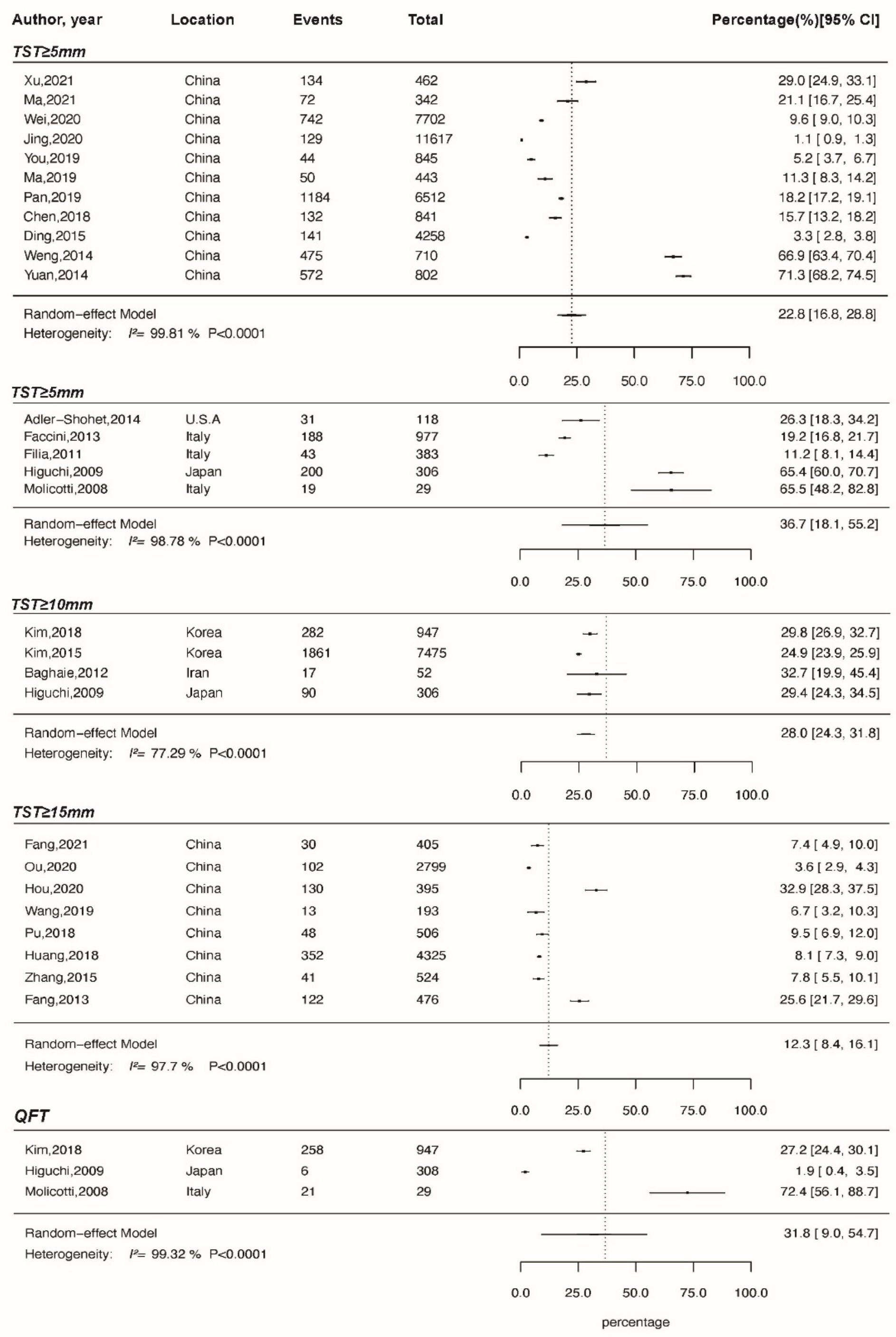

Supplementary Figure 4

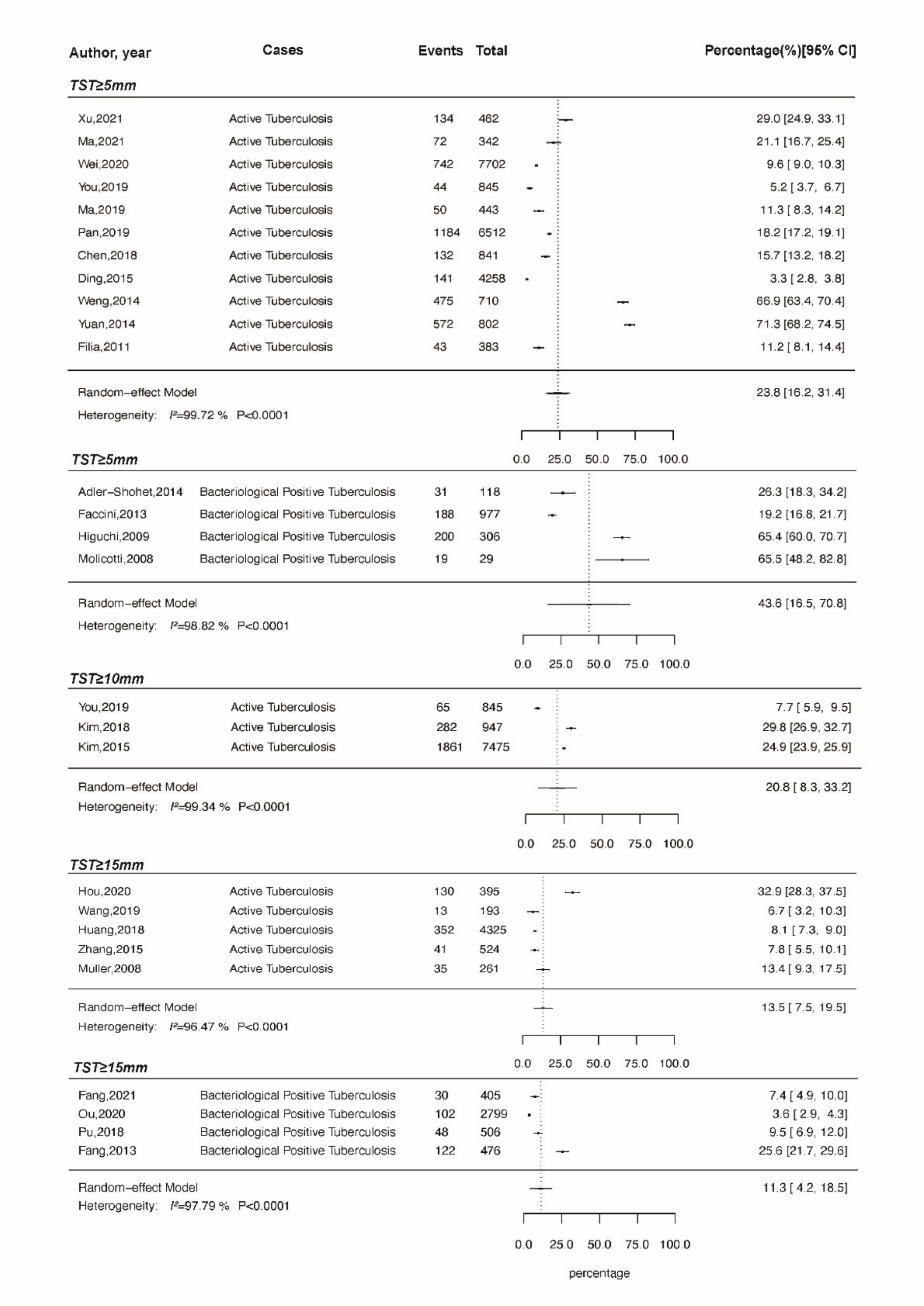

Supplementary Figure 5

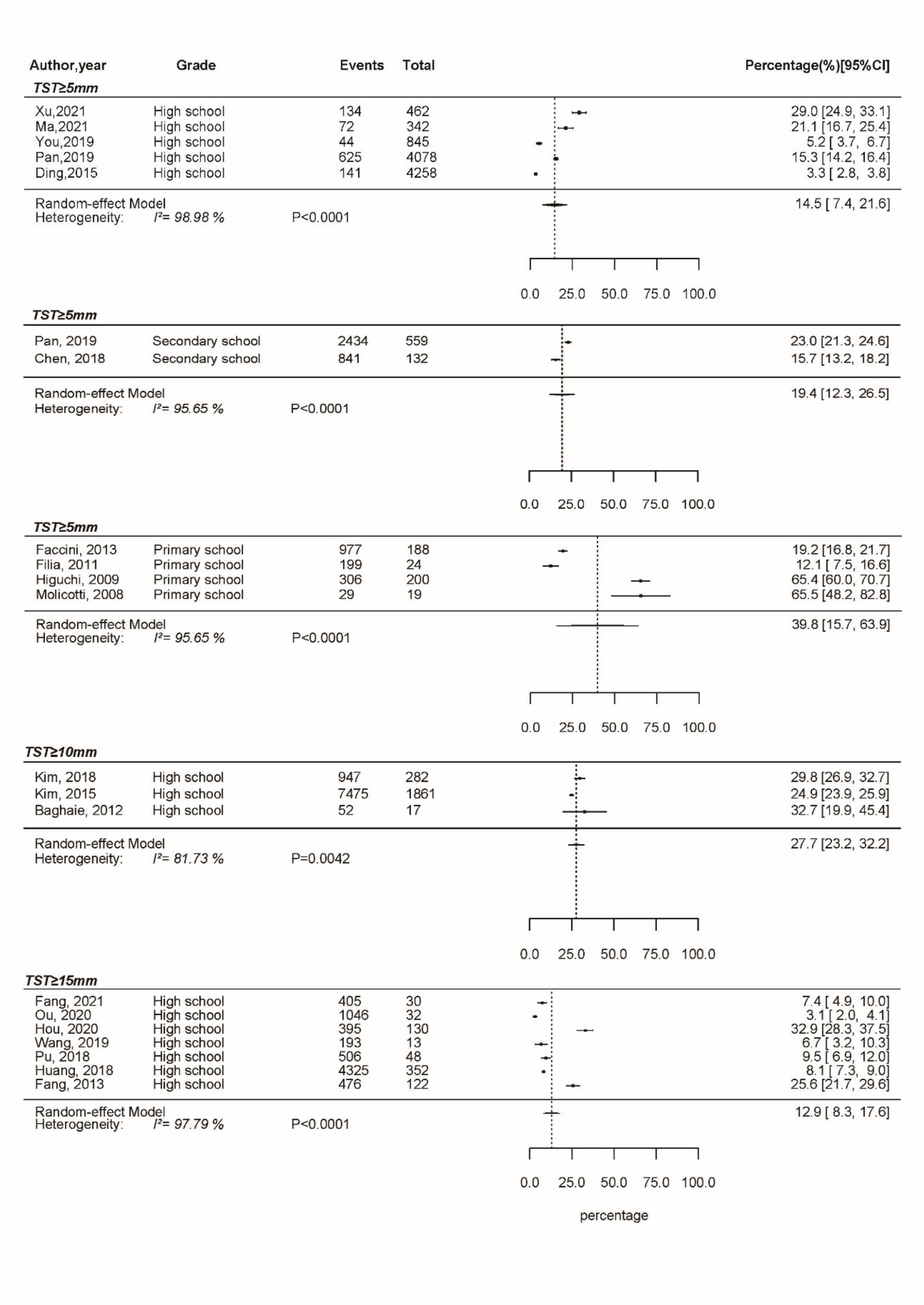

Supplementary Table 1

Number	Author, Year	Level of school	The total number of contacts	The number of contacts screened
1	Adler-Shohet, 2014	Kindergarten	118	118
2	Baghaie, 2012	High school	52	20
3	Chen, 2018	Middle school	N/A	87
			851	841
4	Ding, 2015	High school	4318	4258
5	Faccini, 2013	From primary school to middle school	989	977
6	Fang, 2013	High school	N/A	476
7	Fang, 2021	High school	N/A	52
			N/A	141
			N/A	253
			N/A	193
8	Filia, 2011	Kindergarten、Primary school	388	371
9	Higuchi, 2009	Primary school	313	TST:306，QFT：308
10	Hou, 2020	High school		395
11	Huang, 2018	high school	4724	4325
				156
				206
12	Jing, 2020	Middle school、High school	11617	11488
13	Kim, 2015	Middle school、High school	7475	5714
				1154
				607
14	Kim, 2018	High school	947	947
				632
				24
				258
15	Ma, 2019	Middle school	459	443
16	Ma, 2021	High school	N/A	342
17	Molicotti, 2008	N/A	N/A	N/A
18	Müller, 2008	Other	272	261
19	Ou, 2020	N/A	N/A	N/A
20	Pan, 2019	Middle school、High school	N/A	6512
21	Pu, 2018	VocatioN/Al high school	N/A	506
22	Wang, 2019	VocatioN/Al high school	N/A	1466
23	Wei, 2020	Middle school、High school	N/A	7702
24	Weng, 2014	Middle school、High school	900	179

25	Xu, 2021	High school	N/A	40
			N/A	336
			N/A	79
26	You, 2019	High school	N/A	74
			N/A	496
27	Yuan, 2014	N/A	N/A	N/A
28	Zhang, 2015	Middle school、High school	N/A	524

ATB prevalence(%)	ATB incidence(%)	Sex	Follow-up time	Diagnostic Tests
/	0	/	2 years	TST
15	/	Girls	N/A	TST
2.3	/	One boy/one girl	N/A	TST
/	0.1	Girls	N/A	
0.1	/	N/A	N/A	TST
1.5	/	N/A	One month	TST
/	0.6	N/A	One year and two months	TST
7.7	/	N/A	N/A	TST
/	2.1	N/A		
/	2	N/A		
/	3.6	N/A	Two years and nine months	
5.1	/	Ten boys/Nine girls	N/A	TST
/	0	/	Three years	TST、QFT
2.5	/	One boy/Nine girls	N/A	TST
0.2	/	N/A	N/A	TST
/	1.3	Fifteen boys/Seven girls	Two years	
/	9.7		Two years	
0.3	/	N/A	N/A	TST
/	0.8	N/A	22360 person years	TST
/	2.9		4504 person years	
/	3.5		2355 person years	
2.3	/	N/A	N/A	TST、QFT
/	0	/	3.9 years	
/	0	/		
/	2.7	Six boys/One girl		
1.6	/	N/A	N/A	TST
2.3	/	N/A	N/A	TST
N/A	N/A	N/A	N/A	TST、QFT
1.2	/	Boys	N/A	TST
N/A	N/A	N/A	N/A	TST
0.7	/	N/A	N/A	TST
3.4	/	Boys	N/A	TST
0.3	/	N/A	N/A	TST
0.4	/	N/A	N/A	TST
/	12.9	N/A	One year	TST

0	/	/	Two months	TST
/	0.6	N/A	Six months	
/	1.3	N/A	Nine months	
9.5	/	N/A	13 days	TST
/	1	N/A	24 days	
N/A	N/A	N/A	N/A	TST
0.4	/	N/A	N/A	TST

Bacteriological results
/
N/A
N/A
N/A
Negative
N/A
Three positive
N/A
N/A
Four negative/one positive
N/A
N/A
/
Five negative/five positive
N/A
Negative
Nine positive
N/A
N/A
N/A
N/A
N/A
/
/
Five negative/two positive
Four negative/one positive
Negative
N/A
One positive
N/A
Nine positive
N/A
Negative
N/A
N/A

/
One negative/one positive
N/A
One negative
N/A
N/A
Negative
